# Dimensional changes of peri-implant tissue following immediate flapless implant placement and provisionalization with or without xenograft in the anterior maxilla: a study protocol for a randomized controlled trial

**DOI:** 10.1186/s13063-022-06918-1

**Published:** 2022-11-26

**Authors:** Mehrnoush MeshkatAlsadat, Ali Hassani, Tahereh Bitaraf, Salar Chaychi Salmasi

**Affiliations:** 1 Dentistry Student, Dental Faculty, Tehran Medical Sciences, Islamic Azad University, Tehran, Iran; 2 DDS, MS; Professor, Department of Oral and Maxillofacial Surgery, Dental Implant Research Center, Dental Faculty, Tehran Medical Sciences, Islamic Azad University, Tehran, Iran; 3 DDS, PhD; Assistant Professor, Dental Implant Research Center, Dental Faculty, Tehran Medical Sciences, Islamic Azad University, Tehran, Iran

**Keywords:** Immediate implant, Dental implant, Bone graft, Dimensional change, Buccal gap, Cone beam computed tomography (CBCT)

## Abstract

**Background:**

Dental implant therapy requires the preservation of peri-implant tissue in the cosmetic zone. Various surgical procedures have been presented, including ridge preservation, flapless method, and quick provisionalization. The goal of this research was to assess the buccal bone dimensional changes following immediate flapless implant implantation in the front maxilla, with or without xenografting.

**Method and design:**

Thirty patients who meet the inclusion criteria and have maxillary teeth (numbers 14 to 24) are candidates for the immediate implant with immediate provisionalization. Participants will be assigned randomly to one of two groups: (1) an immediate implant with xenograft and (2) an immediate implant without bone grafting. For 3 months, the temporary prosthesis will be installed shortly before the final restoration. Following temporary prosthesis insertion and 6 months after surgery, a CBCT radiograph will be used to examine bone tissue. Soft tissue will be assessed at three points: baseline, 3 months, and 6 months following implant therapy. Patients’ satisfaction, implant failure, prosthesis failure, and complications will be assessed as secondary outcomes after 6 months.

**Discussion:**

The outcomes of this randomized clinical research will show if buccal bone augmentation with xenograft reduces vertical bone and gingiva recession. The findings and patient-reported outcomes will aid in the selection of therapy alternatives for implant treatment patients.

**Trial registration:**

Iranian Registry of Clinical Trials IRCT20211119053106N1. Registered on 6 December 2021 and Open Science Framework (OSF) on May 20, 2022. Registration DOI 10.17605/OSF.IO/VUGFQ.

## Introduction

### Background

Implant placement is one of the most widely recognized, successful, and dependable tooth replacement solutions. Faster protocols compared to conventional dental implant procedures have been developed because of increased patient desire for cosmetics and social activities. Non-functional loading techniques and immediate implant insertion in fresh extraction sockets have become common therapy, especially in the cosmetic zone [1–3]. Following tooth extraction, bone tissue loss (40% of height and 60% of width) [3] and soft tissue alterations may cause functional and cosmetic issues. Because of its thinness and the existence of bundle bone, the buccal plate is more prone to volume and size decrease. For bone volume maintenance, many procedures such as atraumatic tooth extraction, flapless treatment, and preservation of the residual ridge have been recommended [3]. Many advantages of immediate implant placement (implantation at the same time instantly after extraction into the extraction socket) have been addressed, including preservation of alveolar bone, improved implant durability, psychological benefits, and reduced treatment time for patients [3, 4]. The large buccal gap/surrounding the immediate implant might occasionally interfere with bone repair and osteointegration [5]. As a consequence, excessive bone and soft tissue recession are caused by bone volume decrease and bone defects due to the significant activity of osteoblasts. Previous investigations have shown that small gaps heal naturally without the need for repair [5–9]. There are still some disagreements, and some studies recommend bone grafting if the buccal gap is more than 2 mm horizontally. Others, on the other hand, claim that there is no meaningful difference between utilizing a bone transplant and not using one [5]. A few studies have looked at filling the buccal gap with bone grafting material to minimize bone resorption and gingival tissue height and thickness between the immediate implant and the residual buccal wall [7, 10].

### Objective and hypothesis

The main objective of the randomized control trials is to evaluate dimensional changes of buccal bone after immediate flapless implant placement and bone grafting (xenograft) in the esthetic zone with a buccal gap > 2 mm. Soft tissue dimension changes, patient satisfaction, implant failure, prosthesis failure, and complications as secondary objectives will be examined. The null hypothesis is that xenografting may considerably reduce the recession of peri-implant tissue and increase patient satisfaction.

## Methods

### Overview

**Fig. 1:**
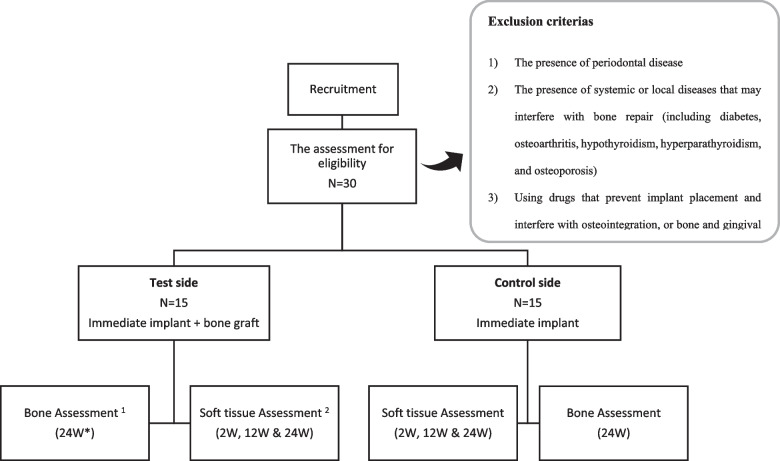
Flow chart of the treatment process. *W weeks. ^1^Using CBCT. ^2^Using an intraoral scanner

**Fig. 2:**
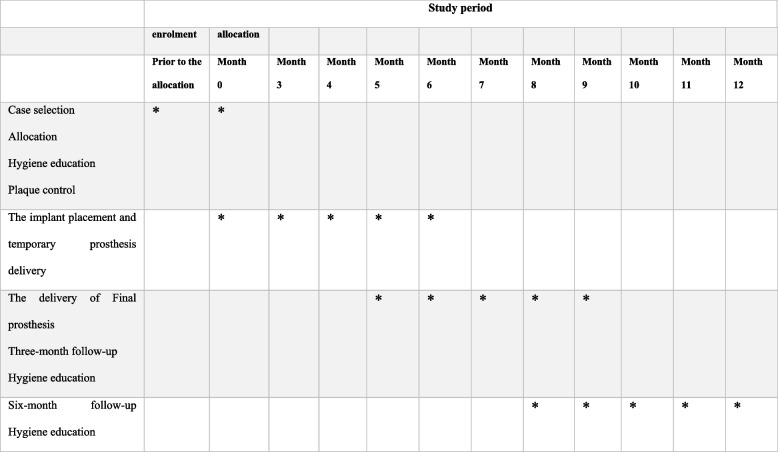
SPIRIT figure

The initial research was planned as a randomized controlled trial. The goal was to enroll 30 patients who needed anterior maxillary teeth and first maxillary premolar dental replacements and evaluate the interventional impact before and after treatment between the intervention and control groups (Figs. 1 and 2). Patients referred to a private clinic and the Oral and Maxillofacial Surgery Department at the Islamic Azad University of Tehran, Iran, as well as a private clinic will be chosen. These two facilities will handle all procedures, follow-ups, and analyses. The Iranian Registry of Clinical Trials (Identifier No. IRCT20211119053106N1) and Open Science Framework (OSF) (Registration DOI 10.17605/OSF.IO/VUGFQ) have been notified about this project. The current study’s recruiting and treatment methodology were authorized by the Research Ethics Committee of the Islamic Azad University-Dental Branch, Tehran, Iran (IR.IAU.DENTAL.REC.1400.096). Before creating a treatment plan, researchers will assess each participant’s overall health and periodontal status. Consent to participate in the research is obtained when the patient’s clinical and periodontal health condition has been established.

### Inclusion criteria

The inclusion criteria for this research are as follows:

1. Patients need the extraction of a maxillary tooth (numbers 14–24) and the possibility of immediate placement of implants on the same site
2. Intact buccal bone [1, 2, 5]
3. Gap space of more than 2 mm between the implant and buccal bone [3, 5]. Immediate implants, if possible, will be placed in the ideal three-dimensional position immediately after tooth extraction, considering the first stability and the possibility of immediate temporary prosthesis placement

### Exclusion criteria

The exclusion criteria for this research are as follows:

1. The presence of periodontal disease
2. The presence of systemic or local diseases that may interfere with bone repair (including diabetes, osteoarthritis, hypothyroidism, hyperparathyroidism, and osteoporosis)
3. Using drugs that prevent implant placement and interfere with osteointegration, or bone and gingival tissue repair (including bisphosphonates)
4. Pregnancy
5. Drug addiction or smoking
6. Acute infection at the implant site
7. Patient undergoing radiotherapy
8. Having parafunctional habits like bruxism or clenching [1, 4, 5]

Patients who fulfill these requirements will be excluded from the research. Selected individuals will have their teeth extracted and will get an implant as well as a temporary prosthesis right away.

### Recruitment

Patients with anterior maxillary teeth and first maxillary premolars (numbers 14 to 24) who need an extraction and are eligible for an immediate implant with provisionalization [3, 11]. Between 2021 and 2022, patients are sent to the Oral and Maxillofacial Surgery Department, School of Dentistry, Islamic Azad University of Tehran, Iran, and a private clinic. The Oral and Maxillofacial Surgery Department is led by A.H and T.B, with S.CS and M.M assisting in recruiting and ensuring approval. Eligible patients will obtain written informed consent that explains the study’s course in straightforward words. The examiners will provide prospective study participants verbal explanations of the research backdrop, research methodology, participation duties, potential risks, and confidentiality regulations. Participants in research may ask questions about the study and are entitled to get all trial information. Before enrolling in the research, each patient must sign an informed consent form after verifying that they understand the study’s goal. They will join the research after indicating their willingness to participate.

### Allocation and randomization

#### Randomization

Each subject will be issued a numerical code that will be placed in a matching envelope that will be kept sealed until the procedure by external investigators. As a result, participants will be divided into two groups: group 1 (with bone graft) and group 2 (without bone graft). Using a web-based randomization service, an independent investigator will produce the randomized sequence (www.randomization.com). Balanced with a block size of 4, blocked randomization will be applied. Only after the implants have been implanted on the day of surgery will the surgeon open the sequentially numbered, sealed envelopes.

#### Blinding

It is impossible to blind the operator. The treatment assignment will be hidden from the data assessor. Furthermore, all patients will be unaware of which group they are in. Operators do not have access to statistical assessment tools due to the principle of blindness. Likewise, statisticians are unaware of treatments and groups.

## Intervention

### Extraction and implant

After extraction in the fresh socket, patients in group 1 (implant with bone graft) and group 2 (implant without bone graft) will receive implants with sandblasted, large grit, and acid-etched (S.L.A) implant surfaces (Implantium, Dentium, South Korea). Using a periotome and forceps, the target tooth is extracted atraumatically with a maximal effort to maintain the buccal plate without flap reflection.

In both groups, implants will be placed 3 mm measured with a millimeter-sized periodontal probe apically to the buccogingival edge of adjacent teeth. The gap width between the immediate implant surface and buccal bone will be measured after implant placement by a periodontal probe to ensure a space of more than 2 mm. Patients with a gap of <2 mm (healed spontaneously without any grafting procedure) will be excluded from the study [5]. After placing the implants, all participants will be divided into two groups: group 1 (implant with bone graft) and group 2 (implant without bone graft).

In patients allocated to group 1, the tooth socket area will be filled with xenograft (SigmaGraft, Fullerton, CA, USA) next to the implant surface after implant insertion. Group 2 will receive an immediate implant that does not need any bone graft material [6]. All surgical operations will be performed by Prof. Dr. Ali Hassani, Oral and Maxillofacial Surgeon.

Prophylactic antibiotics consist of 2 g of oral amoxicillin capsules or 600 mg of oral clindamycin capsules given 1 h before surgery. With the flapless tooth extraction procedure, anesthesia including lidocaine and epinephrine 1/80,000 is injected into the tooth extraction region as infiltration and site preparation [3, 11].

All patients will get the healing abutment for about 2 weeks, once the fixture is installed. Antibiotics (amoxicillin 500mg/clindamycin 300mg) and analgesic (ibuprofen 400mg) will be used to relieve discomfort following implant surgery. The patient will be given chlorhexidine gluconate 0.12% oral rinse (every 12 h for 7 days) after-surgery instructions [3, 11].

We will remove the healing abutment and replace it with a laboratory-modified abutment and a temporary prosthesis that will be non-functional or out of occlusion when the sutures are removed, which is normally 2 weeks following implant placement. The temporary prosthesis will be used for 3 months after the implant is implanted in both groups, following which the permanent prosthesis will be used. To avoid biomechanical stresses from accessing the implant fixture and causing late recovery or treatment failure, the temporary prosthesis should not be functional. The patient will be excluded from the trial if they have parafunctional behaviors like bruxism or clenching [12, 13].

## Outcomes

### Primary outcome (buccal bone changes)

A radiologist will assess bone tissue using a CBCT radiograph created by PlanMeca in Finland in 2016 with the following specifications: voxel size 200 μm, FOV: 8 × 11, 90 kV, and 9 mAs, as well as Romexis measuring software. After the temporary prosthesis is in place, the first tomography examination will be performed. During the examination, a tomographic positioner will be used to standardize the results. To estimate the thickness of the bone at a buccolingual distance, tomographic sections are made such that a slice passes exactly through the center of the fixture. Measurements will be taken apically to the implant platform in the buccal lingual dimension of the slice at 1.5-mm, 3-mm, and 5-mm intervals [3, 5, 11, 14]. In a cross-sectional view and the center of the tooth site, bone height measurements from the buccal crest to the face of implants will be made using Romexis software [15]. Before the procedure, the distance between the buccal bone surface and the implant fixture (GAP) will be measured using a Periodontal Probe CP15 University of North Carolina, and 6 months afterwards by tomographic analysis [3]. All radiographic exams will be performed by an independent examiner [16].

Six months following surgery, a second computed tomography (CBCT) will be performed. With the same basic evaluation methods, the thickness of the bone at a buccolingual distance will be assessed.

### Secondary outcomes

1. Soft tissue changes: A periodontal probe and a stent put on the reference point will be used to assess soft tissue height changes in the mesial, middle, and distal regions of the buccal area of the implant site following implant placement at the surgical visit, 3 months, and 6 months after implant placement [3, 11]. Soft tissue thickness will be assessed at the 4 and 8 mm apically of the mid-buccal gingival margin. It may be done using an 15-endodontic file [11] at 2 weeks, 3 months, and 6 months after implant placement [11]
2. Implant failure: Any implant removed after placement due to mobility, progressive marginal bone resorption, infection, or any mechanical complication during and after the surgery that cannot be forecasted before the procedure (e.g., implant fracture) [16, 17]
3. Prosthetic failure: Any prosthesis which fails with the implant or cannot be placed in terms of implant failure or will be replaced for any reason [16]
4. Biological or prosthetic complications [6]
5. Subjective patient satisfaction of the surgical results: Patient satisfaction esthetically and financially after implant placement [7] will be measured applying three questions, functionally, esthetically, and undergoing a similar treatment again during 1 year of follow-up. It will be performed by possible answers for questions 1 and 2 are “yes, absolutely = 4,” “yes, partially = 3,” “don’t know = 2,” “rather not = 1,” and “Absolutely not = 0.” For question 3, “yes = 1” or “no = 0” [18]

## Sample size

Using the PASS sample size program, the present research sample size was calculated (version 11.0). Paired sample t-test power analysis was used to estimate sample size. If the mean standard deviation of the changes in bone dimensions is 1.2mm and the difference in sizes is 1 mm on both sides, the minimum sample size is 12. The research starts with 15 samples for each group, with a 20% risk of sample dropout [3].

## Data collection and management

Only research team members are allowed to gather data. Members of the study team must keep the information confidential and use it solely for scientific purposes. The information is kept on a secure platform and on paper. Investigators are given a login and password to view the data put into the platform in order to maintain privacy. To eliminate the chance of prejudice, the independent investigator will conduct statistical analysis.

## Training and calibration

The extraction and implant surgical process will be performed by one specialist (A. H) with over 20 years of professional working experience. He is also an expert in the field of rapid implant surgery. Two independent investigators will utilize a periodontal probe to take hard and soft tissue measures before the examination, with the exception of the primary surgeon. Both researchers are dental students with at least 3 years of clinical experience who have been educated in accuracy and reproducibility to assure compliance. Clinical data, patient confessions, and complications will be collected by a certified researcher with sufficient years of expertise to ensure uniformity.

## Statistical analysis

Cohen’s *k* statistic will be used to measure consistency in descriptive data. Frequency numbers (absolute and relative values) and metric data are examples of descriptive statistics (arithmetic mean, standard deviation, and median). The intra-class correlation coefficient will be used to assess consistency between the two examiners for continuous data, such as clinical parameters. The enumerated data will be reported as percentages and the measured data as mean standard deviation or median (quartile spacing). The impact of changes in measurement time between the two study groups at two times (baseline and follow-up) will be compared using an analysis of variance (ANOVA), with the kind of intervention as a between-subject factor. The height and thickness of bone and soft tissue will be quantitative variables. Implant failure, prosthesis failure, comorbidities, and patient satisfaction will all be stated exclusively in terms of frequency (percentage). SPSS 25.0 will be used for all statistical analyses. The significance threshold is specified as 0.05.

## Missing data

The possibility of being untraceable is taken into consideration and incorporated in the computation when evaluating the sample size of the examination. Additionally, missing data (loss to follow-up, death, withdrawal, etc.) will be updated using the multiple imputations approach.

## Ethical consideration

### Ethical approval

The recruitment and treatment protocol of this research was confirmed by The Research Ethics Committees of the Islamic Azad University-Dental Branch, Tehran, Iran (*IR.IAU.DENTAL.REC.1400.096*). Qualified participants will be notified of the consent information of the trial. The ones who are uninterested in signing informed consent will be excluded from the analysis.

### Withdrawal

All patients will be fully informed about the study’s goals and risks prior to any intervention. Patients are able to leave the research at any time for any reason. Regardless of whether or not they participate in the research, all patients will get suitable and effective care.

### Dissemination of result

The findings of the research will be documented and published in a peer-reviewed international publication. The analysis of the research outcomes will be posted at IRCT.ir for public access.

## Oversight

### Trial management

Dental Implant Research Center, Dental Faculty, Tehran Medical Sciences, Islamic Azad University, Tehran, Iran, is leading the present clinical study. The research team will benefit from this coordinating center’s assistance and support in a variety of areas, including trial design, quality assurance, data analysis, dissemination, and study management.

### Steering committee

Several patients and public representatives, as well as two independent doctors and a statistical expert, were recruited to the trial steering group to help guide and oversee the study process. The committee members will meet with the study group every 2 months and are not engaged in the trial’s operation. This committee’s tasks include providing suggestions to the trial research group about the trial’s conduct, data management, monitoring, recruiting, and follow-up methods; statistical analysis of results; and analyzing the trial’s progress to ensure that it follows the study plan.

### Data monitoring

The trial’s progress, adverse events, and data quality will be assessed by an independent Data Monitoring Committee (DMC) made up of members of the Ethics Committee of the Dental Faculty, Tehran Medical Sciences, Islamic Azad University, Tehran, Iran, who are not affiliated with the trial investigators, research team, or sponsors. The research team will meet with an inspector chosen by the DMC on a bimonthly basis to monitor the study’s progress and undertake a temporary analysis. DMC will have the ability to terminate the experiment if any injuries or hazards are discovered, according to the interim findings.

### Harms

There will be no significant injury to research participants, and the surgery method has a minimal probability of failure. The same surgeon will do all of the operations (QL). To reduce the danger of injury, all required precautions will be followed. If something goes wrong, the participants will have a backup restoration plan in place, such as a removable partial denture or a bridge. If a surgical failure occurs together with unanticipated side effects or major adverse events, the DMC will be notified very once.

### Audits

The DMC will have an inspector verify the incoming data every 3 months, independent of the investigators. Each electronic case report form will be checked by the inspector for completion.

## Discussion

Over the last two decades, dental implants have evolved dramatically, opening up new possibilities for tooth replacement that were previously thought to be unattainable [5]. Bone repair employing a mix of reconstructive procedures and rapid implants has shown better outcomes [19]. The space between the buccal bone wall and the implant will be filled with xenograft in this trial [3]. The impact of buccal bone augmentation on the esthetics and shape of immediate implant patients has been studied in many research [20–23]. After implant placement in the socket, bone grafting is often used in the space between the buccal plate and the fixture. It has the ability to maintain the thickness of the buccal bone [24]. Bone grafting in the gap with demineralized bone mineral and 10% collagen following immediate implant placement in locations with an undamaged socket wall greatly decreased changes in the horizontal dimension during implant placement and 16 weeks later, according to Sanz et al. [7]. Tarnow et al. demonstrated bone fusion on the buccal surface of an instantaneous implant implanted in the maxillary canine site with a gap of 4.2 mm, without the need of bone grafting or membranes [25]. Their findings contradict those of Wilson et al., who determined that without the use of membranes, bone healing would be inadequate if there was marginal bone resorption of more than 1.5 mm [26]. This research will look at how employing bone graft material (xenograft) affects soft tissue and bone dimensions in immediate implants in the anterior area when a temporary prosthesis is delivered. This randomized clinical research will determine if xenograft buccal bone augmentation reduces the thickness of the vertical bone and gingival recession. The data and patient-reported outcomes will be helpful in deciding on treatment alternatives for patients who are considering implant therapy.

### Challenges and limitations

Patients who miss their appointments will have a difficult time getting reliable measurements of their buccal bone and soft tissue changes. The true buccal gap declaration during surgery, atraumatic tooth extraction, and flap reflection practice are the key obstacles of this randomized control experiment. The evaluation process, which includes CBCT analysis, should be meticulous.

## Trial status

Recruiting patients is ongoing at the time of manuscript submission (date of submission). Recruitment began on 6 December 2021 and is expected to be completed by 6 December 2022.

## Abbreviation

CBCT: Cone beam computed tomography
